# Feasibility, acceptability, and preliminary efficacy of a self-directed online psychosocial intervention for women with metastatic breast cancer: *Finding My Way-Advanced*

**DOI:** 10.1007/s00520-024-08924-2

**Published:** 2024-10-22

**Authors:** Amy Rigg, Emma Kemp, Bogda Koczwara, Phyllis Butow, Afaf Girgis, Nicholas J. Hulbert-Williams, Billingsley Kaambwa, Riki Long, Penelope Schofield, Jane Turner, Desmond Yip, Robyn Combes, Lisa Beatty

**Affiliations:** 1 Flinders University, GPO Box 2100, Adelaide, South Australia 5001 Australia; 2 Southern Adelaide Local Health Network, Adelaide, South Australia Australia; 3 University of Sydney, Sydney, NSW Australia; 4 University of New South Wales, Sydney, NSW Australia; 5 Edge Hill University, Ormskirk, Lancashire, England UK; 6 Breast Cancer Network Australia, Camberwell, VIC Australia; 7 Department of Psychology and Iverson Health Innovation Research Institute, Swinburne University, Melbourne, VIC Australia; 8 Health Services Research and Implementation Sciences, Peter MacCallum Cancer Centre, Melbourne, VIC Australia; 9 Department of Oncology, Sir Peter MacCallum, The University of Melbourne, Parkville, VIC Australia; 10 University of Queensland, Brisbane, QLD Australia; 11 Australian National University, Australian Capital Territory, Canberra, Australia; 12 The Canberra Hospital, Australian Capital Territory, Canberra, Australia; 13 Consumer Representative and Volunteer, Flinders Centre for Innovation in Cancer, Adelaide, South Australia Australia

**Keywords:** Online intervention, Self-guided, CBT, QOL, Distress, Metastatic breast cancer

## Abstract

**Purpose:**

Few digital interventions target patients with advanced cancer. Hence, we feasibility-tested *Finding My Way-Advanced (FMW-A)*, a self-guided program for women with metastatic breast cancer.

**Methods:**

A single-site randomised controlled pilot trial was conducted. Participants were recruited through clinicians, professional networks, and social media and randomised to intervention or usual-care control. Participants were randomly allocated to either the intervention (*FMW-A;* a 6-week, 6-module CBT-based online self-directed psychosocial program for women with MBC + usual care resources) or control (usual care resources: BCNA’s Hope and Hurdles kit). Feasibility outcomes included rates of recruitment, uptake, engagement, and attrition. Distress, QOL, and unmet needs were evaluated for signals of efficacy, and qualitative feedback was collected to assess acceptability.

**Results:**

Due to COVID-19 and funding constraints, the target recruitment of 40 was not reached (*n* = 60 approached; *n* = 55 eligible; *n* = 35 consented). Uptake was high (*n* = 35/55; 63.6%), engagement modest (median 3/6 modules per user), and attrition acceptable (66% completed post-treatment). Efficacy signals were mixed: compared to controls, *FMW-A* participants experienced small improvements in fear of progression (*d* = 0.21) and global QOL (*d* = 0.22) and demonstrated a trend towards improvements in cancer-specific distress (*d* = *0.13*) and role functioning (*d* = 0.18). However, *FMW-A* participants experienced small-to-moderate deteriorations in general distress (*d* = 0.23), mental QOL (*d* = 0.51), and social functioning (*d* = 0.27), whereas controls improved. Qualitatively, participants *(n* = *4)* were satisfied with the program, perceived it as appropriate, but noted some sections could evoke transient distress.

**Conclusion:**

The study demonstrated feasibility (high uptake and acceptable retention) and generated realistic recruitment estimates. While *FMW-A* appears promising for targeting cancer-specific distress and fear of progression, the mixed findings in quality of life and general distress warrant further revisions and testing.

In the past 15 years, there has been a surge of clinical and research interest in the online provision of self-directed psychological interventions for people with cancer [1], to manage distress [2, 3], anxiety, and depression [4], fear of recurrence [5], quality of life and health service utilisation [6]. However, none has targeted the needs of women with metastatic breast cancer, despite this population being highly prevalent [7], negatively impacted [8], and underserviced. Approximately 35–43% of women with MBC experience impaired QOL and clinically elevated distress [8, 9], similar to those with early-stage disease [9, 10]. However, women with MBC face several unique challenges, such as fear and uncertainty regarding the future, facing mortality, a loss of control, an impact on their sense of identity, reprioritisation of values or goals, dealing with constant or changing treatment schedules, toleration of lifelong side effects of treatment, acceptance of stable disease as a desirable treatment outcome, acceptance of a progressive loss of functional ability, fear of a loss of independence or dependency on others, social isolation, and communicating with others about their illness and mortality [11, 12].

While some of these challenges may overlap with the experience of those with early-stage disease, they are often intensified for women with MBC [12]. Breast Cancer Network Australia’s (BCNA) survey of 582 people with MBC demonstrated three of the most prevalent unmet needs for this population were psychological [13], including the following: (1) fear about cancer progression, (2) fatigue, and (3) uncertainty about the future. If left untreated, impaired QOL and elevated distress are associated with increased mortality rates [14, 15], symptom severity [16], and health service burden [17]. Considering these findings, psychological therapies for this population that target well-being are essential, yet interventions are lacking [18, 19].

Of the few psychological interventions that have been developed for women with MBC, face-to-face group interventions have the strongest evidence base but experience many access barriers [20–22], including being most time-consuming/burdensome and consequently associated with the lowest uptake and adherence [8]. Online self-directed interventions may help to overcome these access barriers, offering anonymity and convenience, where participants can return to the program at any time, potentially facilitating learning and retention [23, 24]. Indeed, in BCNA’s survey, respondents expressed a preference for self-directed strategies to address unmet needs [13].

Our team [25] was among the first to develop an online self-directed intervention for women with MBC, titled *Finding My Way-Advanced* (*FMW-A*). Co-designed with women with MBC [6], the program was developed and refined following feedback from mixed-method scoping studies and think-aloud usability testing [25]. The current study was conducted to evaluate the feasibility and acceptability of *FMW-A (*primary aims) and to explore any signals of efficacy (secondary aim).

## Method

### Design

A single-centre, single-blind parallel RCT, with 1:1 randomisation at the participant level was conducted. The trial adopted the CONSORT guidelines for pilot RCTs [26]. This trial was prospectively registered with the Australian New Zealand Clinical Trials Register (ACTRN12618001492246), and no protocol was published. Ethics approval was obtained from the Southern Adelaide Clinical Human Research Ethics Committee (OFR 258.18), in accordance with the NHMRC *National Statement on Ethical Conduct in Human Research*.

### Settings

Participants were recruited via clinicians at one tertiary cancer treatment centre, social media, and community network advertisements. All RCT participation occurred via a website (www.findingmywayadvanced.org.au) hosted by Flinders University.

### Participants

Participants were as follows: (a) females, (b) aged18 + years, with (c) stage IV breast cancer, (d) a life expectancy of at least 6 months (based on referring clinician’s judgement), and (e) sufficient English for informed consent and program comprehension. Participants were excluded if they had no internet access or active email address or had a previous medical history of dementia or cognitive impairment. A target sample of 40 was selected (20 per arm), as per recommended guidelines for pilot RCTs with small-to-moderate anticipated effect sizes [27].

### Recruitment

Participants were recruited via direct approach by cancer clinicians at Flinders Centre for Innovation in Cancer, professional networks (e.g. Breast Cancer Network Australia’s Review and Survey Group; Cancer Council SA), and sponsored social media advertisements/organic social media posts. Participants who were recruited via social media self-screened for eligibility (and checked a box to state that they meet inclusion criteria) as part of sign-up.

### Procedure

Eligible and consenting participants were emailed a website link to complete baseline-validated self-report measures. They were then randomised 1:1 to either the intervention (*FMW-A* + usual care resources) or control (usual care resources: BCNA’s Hope and Hurdles kit) using an automated computer program. Intervention participants had immediate access to FMW-A and were provided with a PDF tutorial for how to use the program, while all participants were mailed out the usual care resources. Six weeks post-baseline, participants were sent online follow-up measures via an automated email link. As per the Consolidated Standards of Reporting Trials (CONSORT) guidelines [26], concealment was ensured through the separation of data collectors and analysts. Those assessing outcomes were blinded to intervention-assignment.

### *Intervention (FMW-A* + *usual care)*

**Fig. 1:**
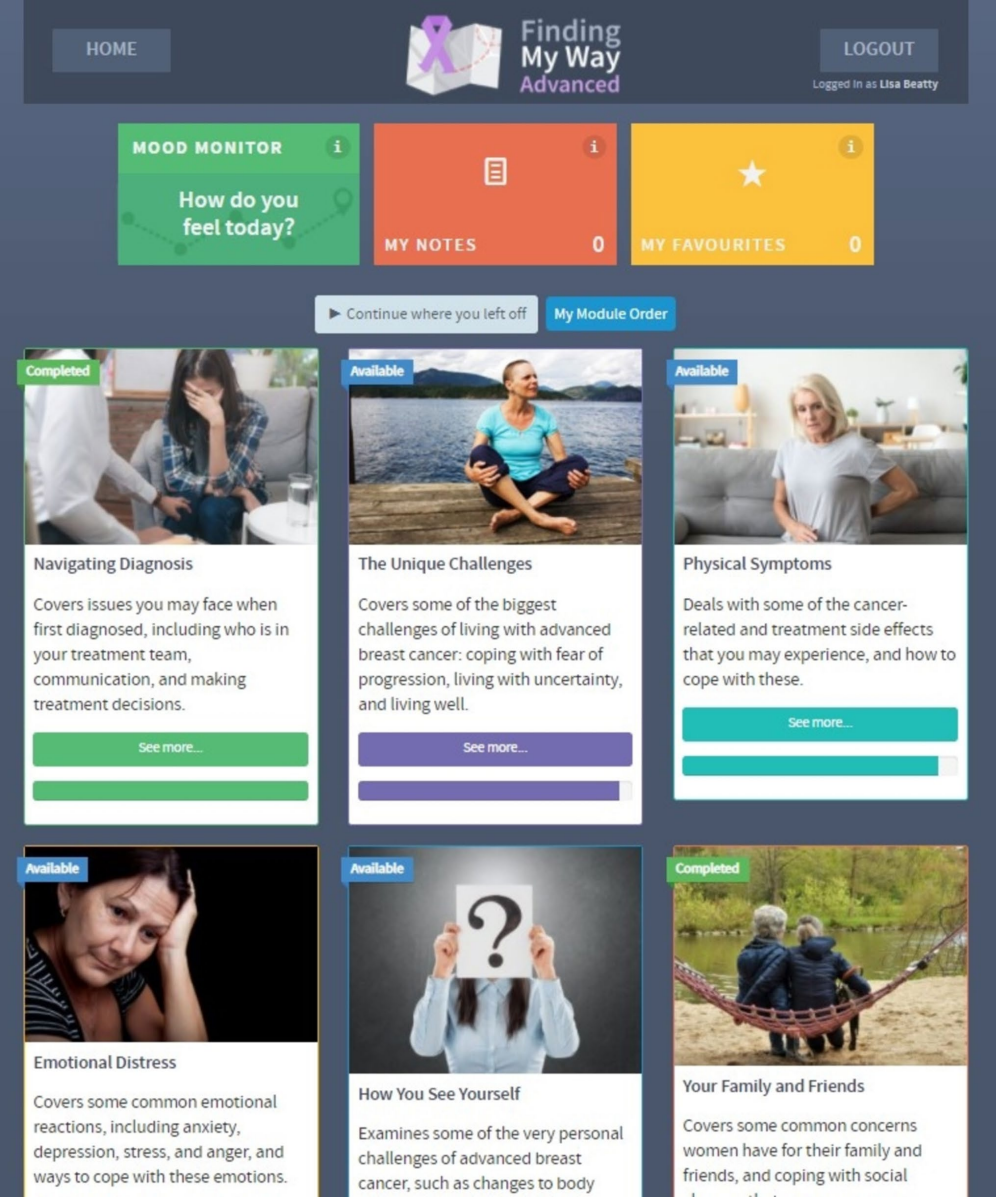
User dashboard

*FMW-A* is a 6-week, 6-module CBT-based online self-directed psychosocial program for women with MBC [25]. The six modules were released weekly, with an accompanying automated email reminder, and cover commonly experienced issues/concerns that arise after diagnosis, namely (i) *Navigating Diagnosis* including communicating with the healthcare team and decision-making; (ii) *The Unique Challenges* of advanced cancer, including managing uncertainty, fear of progression, and planning ahead; (iii) managing *Physical Symptoms*, particularly pain, fatigue, and sleep difficulties, among other side effects; (iv) *Emotional Distress*, including depression, anxiety, stress, and anger; (v) *How You See Yourself*, which covers intimacy, identity, and role changes; and (vi) *Your Family and Friends*, which covers some of the social support concerns women experience and concerns they may have for their loved ones (see Fig. 1 for User dashboard). Within each module, psychoeducation is provided about common symptoms and concerns, along with interactive cognitive behavioural and mindfulness-based worksheets/strategies, and lived experience accounts from other women with MBC. A final booster module is provided 10 weeks after enrolment, providing psychoeducation regarding maintaining well-being, a summary/review of the program, and links back to key resources. Content is presented in various multimedia formats, including text, audio clips, and videos. While module access was tunnelled (limited to one per week), participants could choose/rearrange the module order, and utilise ‘favourites’ and ‘notes feature’ to highlight personally relevant content. Furthermore, based on the qualitative feedback from consumers during the development and usability testing [25], participants were informed to use the program as much or as little as they like and to choose modules, or content within modules, that was relevant to them. Participants were provided contact details for Lifeline and Cancer Council in the footer of the program and within the information sheet and consent form; they were also able to request contact from the research team at the end of each module if they were in the event they experienced emotional discomfort or distress.

Control participants received usual care, namely BCNA’s Hope & Hurdles kit for people with MBC. The kit includes (1) a main booklet with general information about MBC such as diagnosis, support options, treatments, practical issues, and further resources and (2) optional booklets on various topics such as ‘planning ahead’ and specific information pertaining to location of metastases. Participants were phoned to confirm which optional booklets were appropriate for them and were given a link to access digital downloadable copies of the booklets.

### Measures

Participants self-reported age, marital status, number and ages of dependent children, sexual orientation, occupational status, level of education, ethnicity, annual gross income, and area of residence at baseline. Self-reported clinical factors included comorbidities, date of diagnosis, date of previous breast cancer diagnosis (if applicable), treatments received, and prescribed medications. In addition, participants completed a battery of psychometrically validated patient-reported outcome measures at baseline, and again, 6 weeks post-baseline. Participants were not compensated for the completion of assessments.

The primary outcomes were feasibility and acceptability. Feasibility was defined as the preliminary assessment of the intervention and the determination of its appropriateness for further testing [28]. Indicators of feasibility included *eligibility rates* (% eligible participants of those approached; number of eligible participants per month), *uptake rates* (% consenting participants of those eligible; number of consenting participants per month), *program engagement* (number of modules completed), and *research attrition* (% that completed post-intervention survey, % of those eligible who completed qualitative survey). As per Bowen’s feasibility framework, *acceptability* was defined as the suitability, satisfaction, and perceived appropriateness of program recipients [28]. *Acceptability* was therefore assessed via qualitative feedback interviews with a subset of FMW-A participants. Given the scarcity of digital psychosocial intervention trials specifically in the *metastatic breast cancer* setting, as well as the *heterogenous metastatic* cancer setting [29, 30], it was difficult to establish feasibility benchmarks; therefore, the a priori acceptable uptake and retention estimates were derived from a systematic review of (non-digital) psychotherapeutic interventions for MBC: average uptake rate of 53% (range 19–89%) and average retention rate of 69% (range 40–100%) [8, 31]). Acceptable engagement was set at 49% (the percentage of intervention participants who completed 4 + modules in our previous FMW RCT)[6].

Secondary outcomes comprised a battery of psychometrically validated patient-reported outcome measures, including QOL (global QOL, and five QOL functioning subscales¾ emotional, physical, social, cognitive, and role functioning — of the EORTC-QLQ-C30) [32]; general distress (Depression and Anxiety Stress Scale-21) [33], cancer-specific distress (The Post-Traumatic Stress Scale-Self Report) [34]; fear of progression (The Fear of Progression Questionnaire short form) [35]; and unmet needs (The Supportive Care Needs Survey-Short Form) [36].

### Statistical analyses

Descriptive statistics were performed to summarise recruitment and retention indices and baseline characteristics. Between-group *t*-tests were conducted to evaluate differences between (a) completers and drop-outs and (b) intervention and control at baseline. For changes over time, linear mixed-effects models (LMEM) were conducted to account for missing data to generate means and standard effects. However, inferential statistics are not reported, as they are not appropriate for evaluating intervention effects in small samples. Pre-post between-group effect sizes (Cohen’s *d* [37]) and reliable change indices (RCIs) were instead utilised to document the magnitude of the intervention effect.

All *FMW-A* participants were invited to complete a qualitative sub-study at the conclusion of the sixth module, to ascertain how subjectively satisfactory the intervention was. Data were thematically analysed using established guidelines, with a framework analysis used to code qualitative data into a priori themes of acceptability, demand, and practicality (derived from Bowen’s feasibility framework [28]). Two authors collaboratively coded one data set to create an agreed-upon coding matrix and to manage reflexivity. One author (AR) then completed coding for the remaining data sets. A meeting was held at the conclusion of the coding process to further refine reported themes.

## Results

### Primary outcome: feasibility

**Fig. 2:**
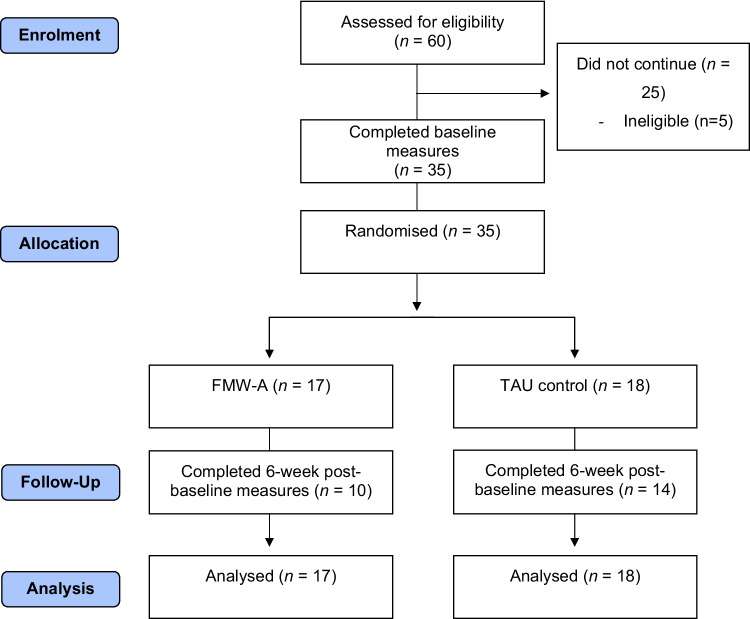
Participant flow through the RCT

Figure 2 summarises the flow of participants through the study. Recruitment commenced 27/2/20. Following the COVID-19 outbreak 2 weeks later, recruitment was paused from March to June 2020 and resumed in July 2020–June 2021. Over the resulting non-extendable 12-month recruitment window, 60 women were referred and assessed for eligibility, of whom 5 were ineligible and 20 declined, resulting in *n* = 35 (*FMW-A n* = 17, control *n* = 18). Thus, the recruitment target of *n* = 40 was not reached; however, the uptake rate was 63.6% (10% higher than the a priori set benchmark), or 2.9 consented participants per month. Uptake rates were similar across recruitment methods (Facebook: *n* = 26 referrals, *n* = 15 consented, 60% uptake rate; Clinicians: *n* = 21 eligible referrals, *n* = 12 consented, 57% uptake rate); however, recruitment efficiency differed (Facebook = 5-week window/*n* = 12 per month; clinicians = 12-month window/*n* = 1 per month).

Table 1 summarises the demographic and clinical characteristics of the sample at baseline. The majority of participants across groups were aged over 55, married, heterosexual, tertiary educated, and identified English as being their first language. Notably, the frequency of employment among *FMW-A* participants was lower than the control group, although this did not reach significance. Over 40% of participants had a de novo diagnosis and were on average 2 years post–MBC diagnosis. There were no significant differences between groups at baseline, except *FMW-A* participants had a higher mean number of medications than controls,$d=1.11$. While there were no significant differences between groups at baseline on any psychosocial outcome variable, inspection of means indicated FMW-A participants were more symptomatic.

**Table 1:** Baseline participant demographic and clinical factors (*n* = 35)

	Control *(n* = *18)*	FMW-A *(n* = *17)*	*p*
Age	56.73 (8.44)	58.42 (10.88)	0.65
Married/partnered	10 (55.6%)	9 (52.9%)	1.00
Heterosexual	17 (94.4%)	16 (94.1%)	1.00
Australian ethnicity	15 (83.3%)	14 (82.4%)	1.00
English first language	18 (100%)	15 (88.2%)	0.23
Employed	9 (50%)	4 (23.5%)	0.24
Gross income < $80,000 > $80,000	9 (50%) 4 (22.3%)	9 (53%) 6 (35.3%)	0.18
Tertiary educated	10 (55.5%)	13 (76.5%)	0.37
Number of children	0.81 (1.05)	0.73 (0.88)	0.82
Mean age of children	17.50 (0.71)	13.50 (9.19)	0.60
Mean time (years) since MBC diagnosis	1.67 (1.40)	2.64 (4.84)	0.16
De novo MBC Mean age of MBC diagnosis mean age of early-stage breast cancer diagnosis Treatments received for MBC Surgery Chemotherapy Radiotherapy Hormonal therapy Other treatment Receiving chemotherapy currently Mean number of prescription medications Other medical condition	7 (38.9%) 56.17 (8.18) 48.18 (9.28) 6 (33.3%) 12 (66.7%) 10 (55.6%) 13 (72.2%) 7 (38.9%) 7 (38.9%) 1.88 (1.31) 10 (55.6%)	8 (47.1%) 54.06 (11.14) 53.56 (12.12) 9 (52.9%) 10 (58.8%) 8 (47.1%) 12 (70.6%) 6 (35.3%) 6 (35.3%) 4.81 (3.56) 12 (70.6%)	0.63 0.53 0.28 0.23 0.32 0.40 1.00 1.00 0.83 **0.006** 0.36

NOTE: bolded results indicate significant effects

The overall study retention rate was met; acceptability thresholds were at 68.6% post-intervention (*n* = 24) but differed by intervention (58.8% intervention group; 77.8% control). One intervention participant formally withdrew (due to other life stressors). Study completers vs non-completers were compared for baseline differences (see Table 2). Completers (*n* = 24) had a significantly higher mean age of early-stage breast cancer diagnosis $t \left(18\right)=2.68, p=0.02 two-sided, d=1.24$ than dropouts (*n* = 11). The intervention group did not predict completion, ${\chi }^{2} \left(1, n=35\right)=0.71, p=0.40, phi=0.20$.

**Table 2:** Demographic and clinical differences between study completers and dropouts

	*Completers (n* = *24)*	*Dropouts (n* = *11)*	*P*
Mean age at baseline	59.11 (9.13)	54.22 (9.74)	0.21
Married/partnered	*20 (83.4%)*	*7 (63.6%)*	*0.21*
Heterosexual	22 (91.7%)	11 (100%)	1.00
Employment status Employed Unemployed Retired	8 (33.3%) 4 (16.7%) 11 (45.8%)	5 (45.5%) 3 (27.3%) 3 (27.3%)	0.67
Gross income < $80,000 > $80,000	11 (45.8%) 8 (33.4%)	7 (63.7%) 2 (18.2%)	0.96
Tertiary educated	21 (87.5%)	6 (54.6%)	0.17
Number of children	0.76 (1.00)	0.80 (0.92)	0.92
Mean age of children	19 (1.41)	12 (7.07)	0.30
Australian	19 (79.2%)	10 (90.9%)	1.00
English first language	23 (95.8%)	10 (90.9%)	0.54
De novo MBC	11 (45.8%)	4 (36.4%)	0.72
Mean age of MBC diagnosis	56.30 (9.90)	52.82 (8.90)	0.33
Mean age of early-stage breast cancer diagnosis	54.69 (10.87)	43 (4.80)	**0.02**
Treatment received for MBC Surgery Chemotherapy Radiotherapy Hormonal therapy Other treatment	10 (41.7%) 15 (62.5%) 13 (54.2%) 17 (70.8%) 11 (45.8%)	5 (45.5%) 7 (63.6%) 5 (45.5%) 8 (72.7%) 2 (18.2%)	1.00 1.00 0.87 1.00 0.30
Receiving chemotherapy currently	10 (41.7%)	3 (27.3%)	0.48
Mean number of prescription medications	3.36 (3.44)	3.30 (2.00)	0.96
Other medical conditions	15 (62.5%)	7 (63.6%)	1.00

NOTE: bolded results indicate significant effects

**Fig. 3:**
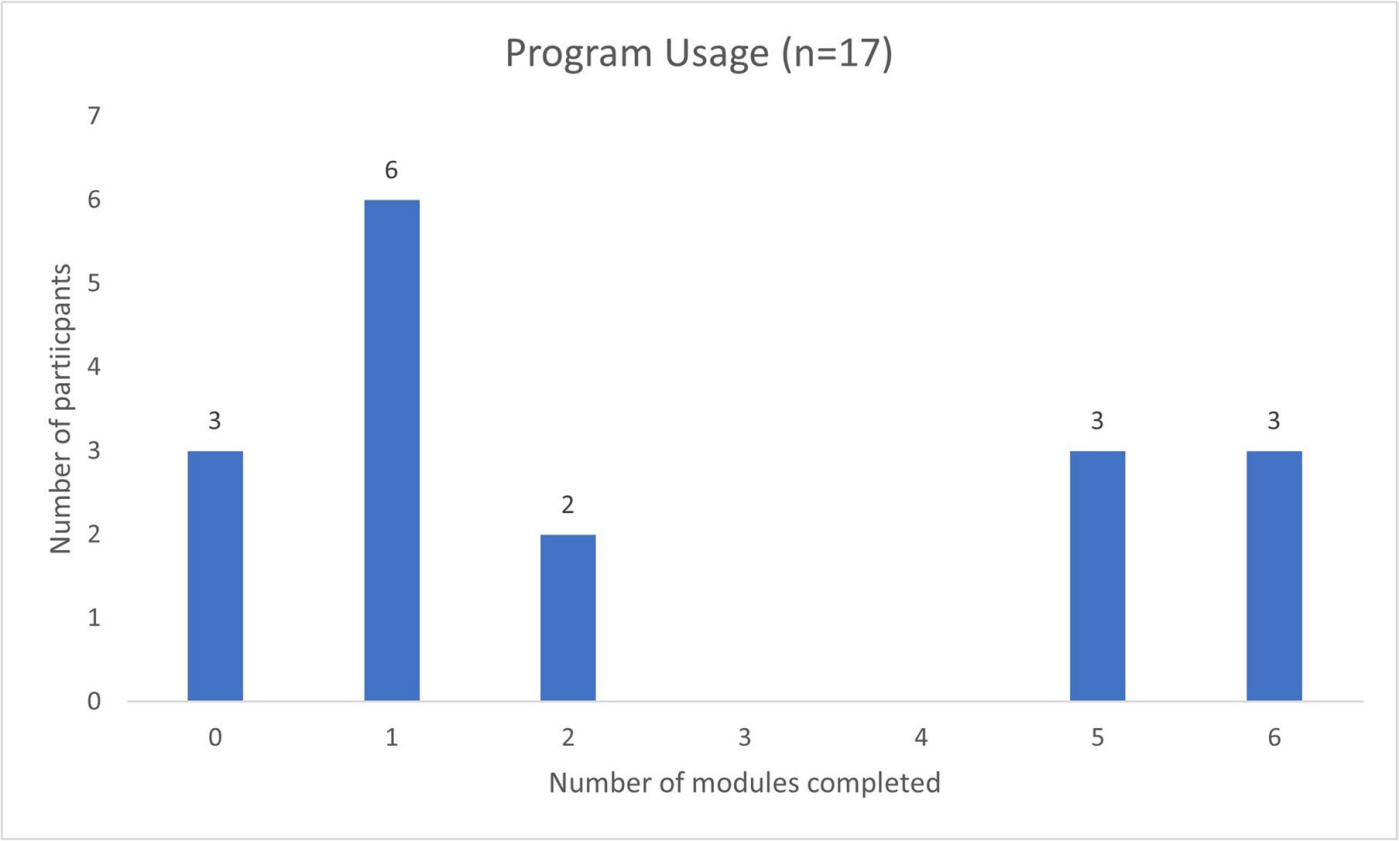
Number of modules accessed by Intervention participants

FMW-A participants accessed a median of 3 modules, with 35% completing the a priori–defined ‘therapeutic dose’ of 4 + modules, thus not meeting the set benchmark of 49%. Figure 3 shows the pattern of module completion. Approximately 18% of participants did not access any modules, 35% accessed 1 module, 11% accessed 2 modules, 18% accessed 5 modules, and 18% accessed all 6 modules. The majority of participants (77%) accessed module 1. Following module 1, there was a large decline in the number of participants who accessed the remaining modules (29% at module 6).

### Secondary outcomes: efficacy signals

Table 3 summarises descriptive statistics, effect sizes, and reliable improvements or deteriorations for all outcomes. Positive outcomes for *FMW-A* were found for four outcomes*: FMW-A* participants experienced improvements in cancer-specific distress $\left(Mdifference=-0.82, d=0.13\right)$ and fear of progression $\left(Mdifference=-2.44, d=0.21\right)$ while control participants’ scores did not change. Similarly, global QOL (*Mdifference* = 4.75, *d* = 0.22) and role function (*Mdifference* = 3.44, *d* = 0.18) showed improvements in FMW-A scores while control participants’ scores did not change. However, in contrast to expectations, *FMW-A* participants deteriorated from baseline to post-intervention on mental QOL $(Mdifference=-5.91)$ and general distress $\left(Mdifference=3.28\right)$ whereas control participants improved $(\text{Mental QOL} Mdifference=5.32$, $d=0.51$; $\text{general distress} Mdifference=-1.25, d=0.23)$. Similarly, *FMW-A* participants deteriorated in social functioning (*Mdifference* =  − 7.28; *d* = 0.27) while controls remained unchanged. Both groups showed a trend towards improved unmet needs from baseline to post-treatment, although this was greater for controls $\left(Mdifference=-8.23\right)$ than *FMW-A* participants ($Mdifference=-2.20$), $d=0.23$.

**Table 3:** Mean, standard errors, Cohen’s *d*, and reliable change indices from baseline to 6 weeks post-baseline

	*Group*	Baseline	Post		Reliable change	
		*M(SE)*	*M*(SE)	*d*	*Improve*	*Deteriorate*
Global QOL *(range 0–100)*	Control FMW-A	57.41 (5.23) 54.41 (5.38)	57.10 (5.66) 59.16 (6.40)	0.22	42.86 50.0	50.0 20
Mental QOL *(range 0–100)*	Control FMW-A	62.96 (5.08) 63.24 (5.23)	68.28 (5.43) 57.33 (6.08)	0.51	42.86 30.0	42.86 60.0
Physical function *(range 0–100)*	Control FMW-A	84.44 (4.48) 72.16 (4.61)	82.11 (4.71) 68.40 (5.18)	0.07	28.6 10.0	50.0 40.0
Role function *(range 0–100)*	Control FMW-A	71.30 (6.49) 57.84 (6.68)	69.59 (7.14) 61.28 (8.22)	0.18	28.57 40.0	35.71 40.0
Social function *(range 0–100)*	Control FMW-A	70.37 (6.41) 55.88 (6.59)	70.49 (6.99) 48.60 (7.99)	0.27	42.86 0	42.86 40.0
Cognitive function *(range 0–100)*	Control FMW-A	68.52 (5.22) 59.80 (5.37)	70.31 (5.61) 60.40 (6.30)	0.05	35.71 30.0	28.57 20.0
General distress *(range 0–42)*	Control FMW-A	27.67 (4.35) 33.41 (4.48)	24.44 (4.90) 34.55 (5.76)	0.23	28.57 30.0	28.57 40.0
Cancer distress *(range 0–51)*	Control FMW-A	13.06 (2.10) 16.00 (2.16)	13.45 (2.28) 15.18 (2.60)	0.13	50.0 50.0	28.57 30.0
Fear of progression *(range 5–60)*	Control FMW-A	36.39 (2.40) 36.71 (2.47)	36.12 (2.55) 34.27 (2.83)	0.21	21.43 40.0	28.57 30.0
Unmet needs *(range 0–170)*	Control FMW-A	79.83 (6.27) 80.82 (6.46)	71.46 (6.75) 78.62 (7.60)	0.23	57.14 30.0	28.57 30

### Qualitative feedback for acceptability, demand, and practicality

All participants were consecutively approached regarding participation in qualitative interviews; however, only 4 participants (one with de novo MBC) consented to participate. The interviews lasted between 15 and 30 min. Interviewed participants were aged between 61 and 77 years.

#### Acceptability

In terms of program *acceptability*, sub-themes included (i) high satisfaction, with participants describing it as a *‘worthwhile activity’, ‘helpful’*, and that they *‘got a lot out of [it]’*, with the lived experience and health care professional videos being particularly valued, and (ii) high perceived appropriateness/fit for women with MBC. Participants noted that different information became relevant across different stages, and two participants indicated that they would have liked to be able to access the program closer to their initial diagnosis. The idea of the program being an additional support was raised by two participants, saying that *‘it reinforced things’* and *‘prompted [them] to start getting back on track’*, or to seek other health services. However, one participant indicated that the information may cause *emotional distress*;

*I really wanted to hear what they had to say but at the same time it was kind of having a little bit of a negative effect on me. I can’t remember which one it was now, I actually had tears, because what she was going through was exactly what I am going through and it hurt, it hurt to hear it.*

#### Demand

There was high perceived *demand* for and interest in the program in the MBC population. The impact of COVID-19 on demand was highlighted. One participant advised they ‘definitely’ would recommend the program to someone else with MBC and to their healthcare team. Actual use of the program was at times lower than planned and impacted by time commitments and symptoms;

*I’ll go back and have a more thorough look in the next little while, but my husband has been away, and I have been here by myself and so my days have been quite full … I’m just tired…*

Mixed intentions to continue use were revealed. One participant stated that *‘[they were] really disappointed when [the modules] stopped’*, while another indicated they would use a similar program again *‘only if it was actually what [they] needed at the time’*.

#### Practicality

The program was described as *‘well set up’* and *‘easy to use’* in general, though one participant found that *‘it was difficult’* navigating the program and advised that *‘anything on the computer is difficult for [them]’*. Participants appreciated that *‘you can go back’* into the modules and found it easy to use in comparison to other self-directed programmes. While some participants had issues remembering *passwords*, these were easily resolved. The weekly release of modules was favoured by some participants as *‘it gave [them] time to catch up with things’* and prevented getting overwhelmed with information, while others expressed a preference to have all modules open to increase access to relevant modules. Several participants discussed how they *‘dipped in and out’* of modules, supporting that the program allowed for personalisation. Two participants commented that the time commitment *‘wasn’t arduous’*.

## Discussion

This study demonstrated the feasibility of an online self-directed psychosocial program for women with MBC, with high uptake rates, acceptable retention, and positive qualitative feedback. While the recruitment target was not met, this pilot has generated estimates for future recruitment timeframes. Although early indicators are promising that *FMW-A* may improve cancer-specific distress, fear of progression, global QOL, and role functioning, this must be balanced against the mixed findings arising in other quality-of-life domains, particularly mental QOL, and the increases in general distress. Given the small sample size, the potential impact of baseline distress on program engagement and subsequent escalations of some symptoms, revisions to the program, and replication of findings are warranted via a larger study before efficacy conclusions are drawn.

Preliminary indicators of feasibility were obtained: uptake was high in the enrolment period at 63.6% — notably 10% higher than the documented pooled uptake rate of MBC intervention RCTs [8] and 20% higher than the average uptake rate for group therapy RCTs. This uptake rate is similar to individual therapy RCTs for MBC [8]; while one might have predicted uptake to be superior in overcoming accessibility barriers, these modalities reach arguably different populations with different needs and preferences [38]; thus, comparable uptake rates are a positive outcome. While the uptake rate was nearly identical for those approached via clinicians vs social media, the recruitment rate per month varied dramatically (1 per month via clinicians vs 12 per month via social media). This was consistent with other recent trials utilising social media advertising to augment recruitment efforts for their digital psycho-oncology trials [39, 40] and highlights the importance of this recruitment avenue. Limitingly, those who were recruited via social media self-screened for eligibility as part of sign-up. Future research may consider obtaining consent from this recruitment group to contact their treatment team to confirm eligibility and medical information.

While uptake was high, twenty eligible individuals declined to participate. In many instances, reasons for declining were unable to be documented but commonly cited reasons for declining related to already coping well and/or having adequate support. Potential other reasons might relate to those arising among participants with lower engagement levels — e.g. higher distress and not wishing to be exposed to content. Alternatively, people with advanced cancer experience time toxicity (i.e. losing time as a result of cancer treatments), causing them to prioritise how they spend their time) [41].

Retention indicators of overall trial feasibility met acceptability benchmarks (attrition rate of 31.4% post-intervention) and were identical to the pooled attrition rate documented in a systematic review of MBC RCTs [8]. This was further demonstrated via qualitative feedback, where participants found the program acceptable, practical, and in demand. Due to the accessibility of the program during Covid-19, there may have been artificially increased demand for the intervention at the time of this trial, though it could equally be argued that people were ‘zoomed out’ during the pandemic and were less inclined towards digital modalities than at other timepoints. Participants also provided positive feedback about the program serving as a prompt to access more intensive services, consistent with findings of increased supportive care service utilisation in the *FMW* trial [6]. However, it should be noted that limited conclusions can be made regarding the qualitative feedback within the current study, due to the small sample size of self-selected participants who provided this feedback that are not necessarily a representative sample of the MBC population. Furthermore, retention after randomisation differed across conditions (77.8% for control versus 58.9% for intervention). Thus, while the overall trial’s retention rate met a priori feasibility benchmarks, the intervention group’s rate was 10% below and thus sub-optimal and may reflect the increased time commitment, higher distress experienced, and higher digital health literacy required. However, qualitative data indicated that the program was *not* viewed as too large of a time commitment. Although, this may be biased, given participants are self-selected for the interview and are likely to be high program engagers. Alternatively, while participants were provided with a PDF tutorial for how to use the program, this may not have been sufficient for those with low computer literacy skills, as indicated to be a potential barrier in the qualitative feedback.

Contrary to the literature supporting that low-intensity interventions have higher adherence than face-to-face psychological interventions [8, 42], programme engagement in the current study was lower than expected at a median of 3/6 available modules; on average completing one module less than the original *FMW* [6]. While the a priori benchmark for engagement was not met, there is limited evidence to suggest what ‘dose’ of digital self-guided interventions is required to derive benefit [43]. Interestingly, the bi-modal pattern of program engagement noted in this study (see Fig. 3), whereby participants tended to complete either very little or nearly all of the program, is common in digital interventions for cancer populations [20, 44], however, seemingly amplified in the metastatic setting. Indeed, these findings may reflect the individual needs of participants, given they were informed to use the program as much or as little as they like and to choose modules, or content within modules, that was relevant to them. Alternatively, this may reflect the nature of cancer generally, given this is a typical finding in digital mental health [2, 6], or reflect the nature of MBC specifically, given the program is targeting those with a chronic condition, whose illness demands fluctuate Future research may consider removing the tunnelling/sequential release of modules, to enable immediate full access. A common theme raised in the qualitative analyses was participants’ preference to *dip in and out* of modules. This fits the adult learning framework proposed by Cornelius and Gordon, whereby there are three types of adult learners¾ *universalists* (who study all available materials), *butterflies* (who dip in and out of materials), and *changelings* (who change their study approach over time) [45]. Thus, the participants in this study may fall into the category of *butterflies.* While this pattern of engagement results in lower overall usage rates, immediate access to all modules may increase usage rates and cater to all three categories of learners. While outside the scope of the current feasibility study, future research may include a longer follow-up period to accommodate people who may have an increasing number of clinical events and needs for which they might seek information via the program.

Furthermore, while digital intervention engagement is considered important for intervention effectiveness in the broad adult population [46], there is mixed research regarding the association between increased engagement and improved outcomes within the *curatively* treated cancer [44] and survivorship populations [47–49]. Therefore, it is critical to understand how those in the advanced/metastatic setting engage with digital interventions and how this engagement is related to psychological outcomes. Future research may (a) aim to increase engagement of those in the advanced/metastatic setting to determine its impact on the efficacy of digital interventions, (b) explore engagement correlates (e.g. demographic characteristics, baseline outcomes), (c) qualitatively evaluate engagement further with a larger sample of participants (i.e. facilitators, barriers, self-reported engagement versus objective indicators of engagement), or (d) modify the intervention to suit participants current level of engagement, through the development of a micro intervention. Some promising signals of efficacy were obtained. While cancer-specific distress, fear of progression, global QOL, and role functioning did not change across time among control participants, *FMW-A* participants mean scores showed trends towards improving. The changes in fear of progression in particular for *FMW-A* participants demonstrated an overall shift from the ‘high’ to the ‘moderate’ range, according to Sarkar and colleagues’ cut-off score of 34 [50]. However, the less promising findings for *FMW-A* participants in unmet needs, general distress, and mental and social QOL from baseline to post-intervention were contrary to expectations and potentially concerning. These aligned with qualitative findings, where one participant discussed how some information in *FMW-A* may cause emotional distress or discomfort. However, a body of literature shows that exposure to potentially emotional stimuli or situations previously avoided can lead to short-term increases in distress, particularly among those more vulnerable to distress, prior to habituation occurring [51–53]. Given that distress is negatively associated with QOL in the breast cancer population, the increased general distress of *FMW-A* participants may be explained by these exposure principles and explain the decreased QOL scores in turn. In addition, the efficacy of the intervention may have been impacted by the lower engagement observed. Future studies need to include a follow-up period longer than 6 weeks to determine if general distress habituates, and as a result, QOL starts to increase. Conversely, this finding could reflect the impact of (dis)engagement and ongoing avoidance of content.

That said, to address the potential that distress was caused by the program, a number of changes could be made for future testing: removing module-tunnelling and unlocking all modules at once (enabling freedom of choice regarding which modules to access), introducing an ‘accordion’ function that opens up further information within modules when clicked (to enable freedom of choice regarding which information within a module participants access), improved worksheet functionality and consequently improved user experience, and the separation of research surveys from the therapeutic program to clearly delineate content and reduce overwhelm. In addition, further studies are underway regarding the addition of human support to the program, aiming to increase engagement, and consequently, outcomes.

Another solution is a modification of *FMW-A* into a blended format, where online-based therapies are used in adjunct to face-to-face therapy or as part of a stepped care model, whereby baseline distress is screened prior to participation to determine the suitability of participants for a self-directed protocol. Indeed, systematic reviews in the mental health population support that online interventions with guidance are more effective in improving engagement and psychological outcomes, such as anxiety and depression [54, 55]. A recent systematic review found that health professionals preferred blended therapy over online only [56], particularly for those with more severe psychological distress who may be at higher risk of dropout and may benefit from the therapeutic rapport and more active monitoring. Previous blended therapy trials in cancer populations have shown effectiveness in improving fear of cancer recurrence [57] and psychological distress [58]. However, these have focused only on those in post-treatment survivorship rather than metastatic populations and have not measured the impact on QOL. *FMW-A* could provide important data for a blended model.

In conclusion, while this study was limited by a small sample size, this is the first randomised trial of an online self-directed intervention for women with MBC, demonstrating the feasibility and acceptability of the program and avenues for improvement. Preliminary signals of efficacy were found but must be balanced against the mixed findings and potential for escalations in distress among those with high baseline symptoms who are less likely to engage with the program. Further research is warranted to revise the program and replicate findings in a larger study and to explore avenues for improving program engagement.

## Data Availability

No datasets were generated or analysed during the current study.
